# The effect of individual and paired Brailletonik exercises on balance and reaction time in children with intellectual disability

**DOI:** 10.1186/s13102-024-00891-9

**Published:** 2024-05-03

**Authors:** Zohreh Janbozorgi, Hasan Khalaji, Jalil Moradi

**Affiliations:** 1 Department of Motor Behavior and Sport Psychology, Faculty of Sport Sciences, Arak University, Arak, 38156-8-8349 Iran; 2 Research Institute of Applied Studies of Sports Sciences, Arak University, Arak, Iran

**Keywords:** Balance, Reaction time, Intellectual disability

## Abstract

**Background:**

children with Intellectual Disabilities (ID) are less proficient in motor skills compared to normally developing children, which means they need more time for learning skills. In this context, the purpose of this research is to investigate the effect of the Brailletonik physical activity program (BPAM) on the balance and reaction time of children with ID.

**Methods:**

The statistical sample is consisted of 30 children aged 8 to 12 (21 boys, 9 girls) with ID with an average age of 9.8 ± 1.39, who were selected through convenience sampling. Participants were divided into two groups of individual BPAM, and pair BPAM. Training sessions were held for 21 sessions (seven weeks) and each session included 30 min of training. To measure static balance and reaction time, Stork Stand test and Simple Reaction Time Software were used respectively. The analysis of covariance (ANCOVA) and Bonferroni’s *post hoc* test were used to analyze the data. Data analysis was performed using SPSS software version 26 at a significance level of *p* ≤ 0.05.

**Results:**

The results showed that individual and paired exercise groups had significant progress from pre-test to post-test in both variables of balance and reaction time (*p* = 0.001). Also, the comparison of the performance of the groups in the post-test showed that the average performance of the paired exercise group was significantly better than the individual exercise group in the balance variable (*p* = 0.03) and in the reaction time variable (*p* = 0.01).

**Conclusion:**

Accordingly, it can be concluded that BPAM in paired groups has a greater effect on the balance and reaction time of children with ID.

## Introduction

Intellectual disability (ID) includes significant limitations in intellectual functioning and adaptive behavior that begin before the age of 18. It has been reported that the prevalence of ID is about 1 to 3% of the world’s population. In Iran, the prevalence of ID is higher among males (5.3/1000) than females (3.5/1000) and the highest rate is for adolescents and young adults [1]. Children with ID have a lower level of skills such as physical strength, motor coordination, running speed, perceptual-motor performance, and balance [2]. Children with ID learn basic motor skills more slowly than normally developing children [3]. One of the important variables in these children is balance. Balance is the ability to maintain or return the body’s center of gravity within a stable range determined by the base of support [4], and is one of those basic skills that are necessary to learn and perform for all ages and levels. As such, in order to establish balance for most functional tasks, the vertical orientation of the body should be maintained [5]. There are two forms of balance: dynamic (body stability in a moving state such as walking and running) and static. Static balance is when the center of gravity remains constant, such as standing or sitting [6]. Of course, this component is different in each individual and many factors such as the age, overweight and obesity, gender, and physical activity level affect it [7]. In a research study, Giagazoglou et al. (2013) aimed to investigate the impact of trampoline training intervention on the motor performance and balance of children with ID. They concluded that trampoline training led to a significant improvement in the performance of the participants in all motor and balance tests, hence its interesting and enjoyable exercises can be used in the improvement of these children [8]. In a meta-analysis study, Ma et al. (2019) investigated the effects of sports programs on the balance ability of children with ID. The results showed that the best outcomes can be achieved with at least 30 training sessions of 45 to 60 min. Also, these exercises showed better results in children aged 5 to 13 than in 14–18-year-old children [9].

In addition to the factor of balance, children with ID are also weak in reaction time (RT) compared to normally developing children [3]. Reaction time (RT) is a measure of how quickly an organism responds to a stimulus. RT is defined as the time interval between stimulus presentation and the subject’s response. RT is an indicator of the speed of decision-making and efficiency, and in many of the fast-motor skills, the success of the athlete depends on the speed with which he can analyze the environmental conditions or the movement of the opponent and then decide on showing the appropriate reaction. RT is classified into three types: simple, choice, and discrimination. RT is simple when the experimenter uses only one stimulus and requires only one response [10]. Factors such as age, number of stimulus-response, compatibility of stimulus-response, gender, physical activity, training, fatigue, and nutrition affect this variable [11–13]. Yıldırım et al. (2010) conducted a study with the aim of investigating whether reaction time can be improved with an exercise program. After 12 weeks of structured physical training, a post-test was taken and the results showed an improvement in reaction time in the experimental group [3]. In another study, Dehghani and Gunay (2015) investigated the effect of balance training on the reaction time of children with ID. As the intervention, the experimental group was trained for 10 weeks in 45-minute sessions. The results showed a significant difference between the experimental group before and after the intervention [14].

A review of the related literature shows that many studies have investigated the effect of different sports programs on balance and reaction time of children with disorders, but one of the important sports disciplines to help children with ID is rhythmic Brailletonik exercise. Movements in this exercise involve balance, coordination, understanding of spatial and temporal relationships, and orientation of all or parts of the body; also, performing movements with happy music enhances children’s interest and participation in the activity [15]. Brailletonik is an exercise based on coded letters and is inspired by the international Braille script; the execution of these movements with hands and feet is based on a six-house table in a visual, numerical, movement and sports form [16]. Brailletonik exercise is related to other sports as well and it can be used in warming up; it is also effective in improving children’s performance in such fields as gymnastics, squash, tennis, basketball, darts, golf, shooting, Frisbee, swimming, boxing, skating and cycling [15]. Regarding the effect of the Brailletonik exercise, Dehghanizadeh et al. (2018) investigated the effect of a course of Brailletonik exercise on the motor skills of children with ID. The results indicated that the Brailletonik exercise is effective in the development of motor skill in children with ID [17]. In another research, Rahmati Arani and Dehghanizadeh (2020) conducted a study with the aim of investigating the effect of a Brailletonik training course on static and dynamic balance and the coordination of children with ID. The results of this research shows the positive effect of Brailletonik training on balance and coordination [18].

Regarding the necessity of conducting this research, several important points can be mentioned. The review of previous studies on the effect of Brailletonik exercises shows that few studies have investigated the effect of these exercises on children with ID. In addition, the effect of these exercises on the important variables of balance and reaction time has not been investigated yet. Furthermore, no research has been done in the field of comparing the effect of Brailletonik exercise individually and in pairs. One of the widely used methods in educational environments is pair training methods. In individual and competitive practice, children experience negative interactions to reach the goal; while in pair training methods, the learner seeks to experience positive mutual relationships, more effort and helping each other to reach the goal. Additionally, the implementation of this research can lead to a better understanding of the factors affecting the improvement of motor performance of children with ID, because balance and postural problems lead to the withdrawal of disabled children from sports activities and daily movements, therefore, providing motor programs to improve this skill is mandatory [19]. Considering the above-mentioned points, the purpose of this research is to investigate the effect of individual and paired Brailletonik exercises on the reaction time and balance of children with ID.

## Methods

### Participants

The research method was semi-experimental, which was carried out with a pre-test and post-test design with a control group. The statistical sample of the research comprised 20 children (21 boys and 9 girls) with ID in the age range of 8 to 12 (mean age of 9.8 ± 1.39) who were selected through convenience sampling. The participants of this research were students of a special school for exceptional children whose intellectual disability was identified by a trusted psychologist as well as a health center at the beginning of their admission to the school. The sample size was estimated based on the G-power software, considering effect size of 0.8, alpha of 0.05, and statistical power of 0.95. The inclusion criteria comprised not having injuries and abnormalities such as blindness, deafness, and physical disability. The exclusion criteria were the individual’s lack of satisfaction and non-cooperation in the research process until the end of the program while observing the symptoms of the COVID-19 disease in each individual. After further explanation about implementing the exercises, written consent was obtained from the students’ parents. The current research was carried out with the approval of the ethical committee considerations for human research at Arak University (IR-ARAKU.REC.1401.048). Demographic characteristics of the participants by groups are presented in Table 1.

**Table 1:** Demographic characteristics of the participants

Variables	Groups	
	Individual exercise (M ± SD)	Paired exercise (M ± SD)
Age (Year)	9.50 ± 1.35	10.50 ± 1.18
Height (cm)	129.20 ± 6.12	135.00 ± 9.64
Weight (Kg)	33.20 ± 8.87	33.70 ± 7.62
Body mass index (BMI)	20.07 ± 6.14	17.74 ± 2.13
Intelligence Quotients (IQ)	58.4 ± 4.92	56.8 ± 6.22

### Research instruments

A) Stork Stand Test: The tool used to check static balance was the Stork Stand Test [14]. In this test, the examiner stands on one leg and the toe, and bends the free leg from the knee, and keeps it close to the hip. Using a stopwatch, 60 s is taken and the time stops whenever the person loses his balance. The number of times a person loses his balance is recorded. If the participant falls more than 15 times (loss of balance) in the first 30 s of the test, the test will be terminated and the subject’s score will be zero. In previous studies, high validity (0.99) and reliability (0.87) have been reported for this test [20, 21].
B) Simple reaction time test: Simple reaction time test software was used to measure the variable of reaction time. This software is used as a CD and uses a visual stimulus to measure simple reaction time in different age groups, so that a colored dot appears on the screen and the person presses the response key on the computer keyboard after presenting the stimulus. This tool has been developed and introduced by Donders, and Kosinski in his study reported the reliability of this test to be 0.79 [22]. In this study, the reaction time software developed by the Sina Institute of Behavioral Sciences was used which was adopted from the Donders instrument [23]. The administration of this test took about 7 min.

### Procedures

First, in a briefing session, the researchers got to know the participants and gave a short explanation about the exercise sessions and how to participate in the research. In the next session, a pre-test was held for balance and reaction time tests. Then, the participants were randomly divided into two groups of 10 members (individual and paired exercise groups). In the individual training group, the movements were performed individually without coordination or attention to another person. In the pair exercise group, participants had to perform their movements in harmony with a partner. The experimental groups participated in Brailletonik Physical Activity Program (BPAM) for 21 sessions every other day. The training sessions lasted for 30 min, including 5 min of warm-up, 20 min of the training program, and 5 min of cooling down. BPAM included: (1) hopping on the Brailletonik table; (2) jumping from one leg to another on the Brailletonik table; (3) jumping with both legs on the Brailletonik table with eyes open after hearing the whistle; (4) jumping with both legs on the Brailletonik table with eyes closed after hearing the sound of the whistle; (5) placing colored balls in the predetermined houses of the Brailletonik table; (6) making a paper ball and throwing it on the predetermined houses of the Brailletonik table; (7) showing a simple word like “water” with jumping on the Brailletonik table after hearing the sound of the whistle; (8) showing a simple word like “water” on the body; (9) showing a simple word like “water” with jumping in space; (10) jumping with one leg on the Brailletonik table after hearing one whistle and throwing the ball towards the basket after hearing two whistles [17].

**Fig. 1:**
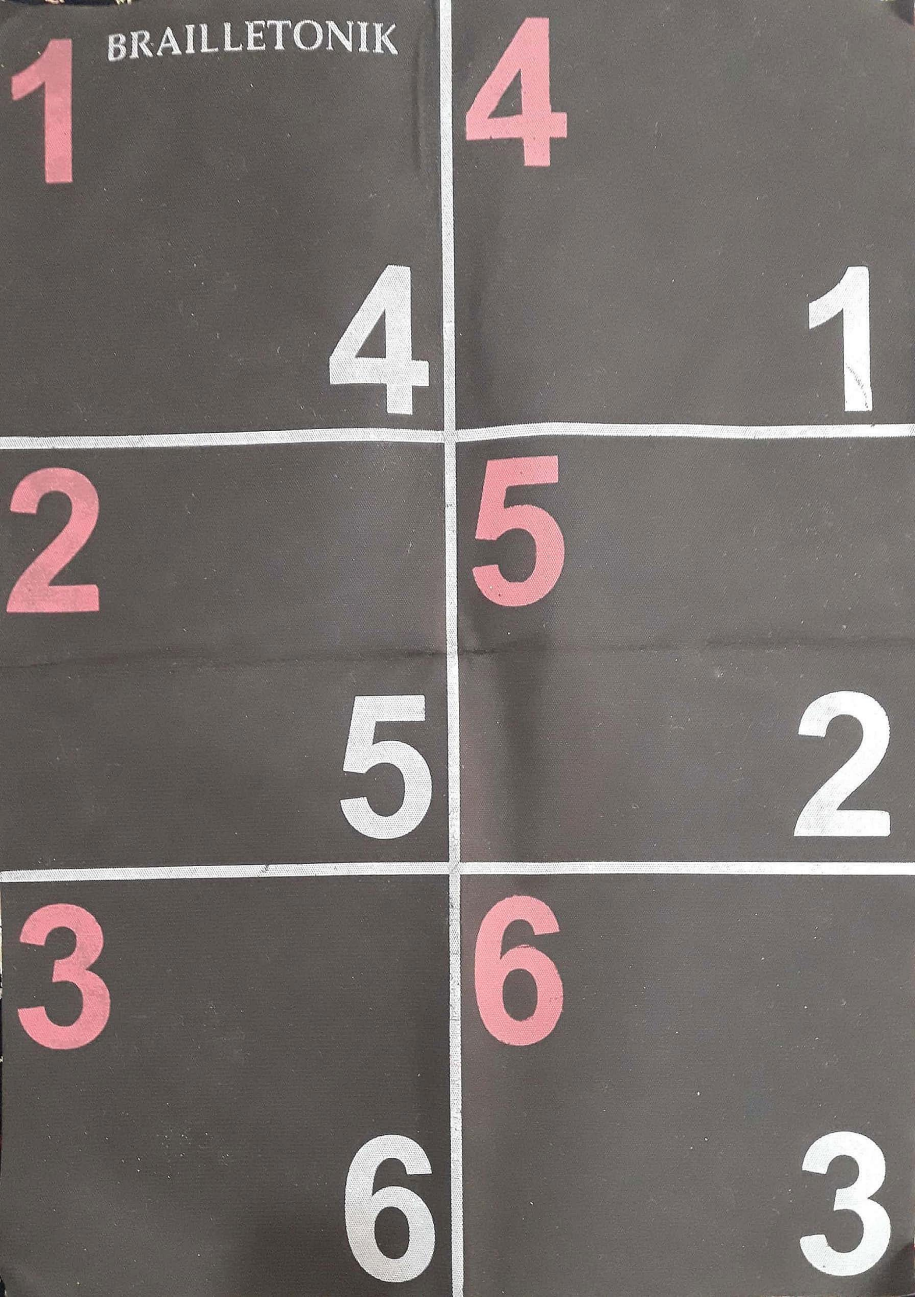
A view of the Brailletonik exercise page

In the exercises with eyes open, the paired groups performed the exercise in coordination with each other, and in the exercises with eyes closed, one of the two children guided his/her partner in the direction of performing the correct movement. At the end of 21 sessions, the post-test of balance and reaction time was performed. Figure 1 shows a view of the Brailletonik exercise page.

### Statistical procedures

The Shapiro-Wilk test was used to check the normality of data distribution. Considering that the scores of the participants of the groups were different in the pre-test, analysis of covariance and Bonferroni’s *post hoc* test were used to analyze the data. The Partial Eta Squared was also used to investigate the effect size. The data were analyzed using SPSS version 26, and the significance level of 0.05 was considered in all statistical procedures.

## Results

The mean and standard deviation of the scores of the participants in the balance and reaction time tests by groups are presented in Table 2.

**Table 2:** The mean and standard deviation of participants’ scores in balance and reaction time tests

Groups	Balance (Number of falls)		Reaction time (ms)	
	Pre-test	Post-test	Pre-test	Post-test
Individual exercise (M ± SD)	18.00 ± 8.69	13.90 ± 7.38	1385.15 ± 515.03	1046.17 ± 419.15
Paired exercise (M ± SD)	16.60 ± 7.53	9.40 ± 6.33	1231.76 ± 207.90	613.35 ± 113.30

As seen in Table 2, in both variables of balance and reaction time, the paired exercise group performed better than the other group in the post-test. After ensuring the normality of the data distribution, (given the difference in the pre-test score) analysis of covariance was used to compare the groups.

The results of the analysis of covariance test showed significant difference between the groups in the balance variable (F_(1,17)_ = 6.91, *p* = 0.01, and η^2^ = 0.28) and the reaction time variable (F_(1,17)_ = 18.83, *p* = 0.001, and η^2^ = 0.52). Bonferroni’s *post hoc* test was used for comparing multiple means in both balance and reaction time variables.

**Fig. 2:**
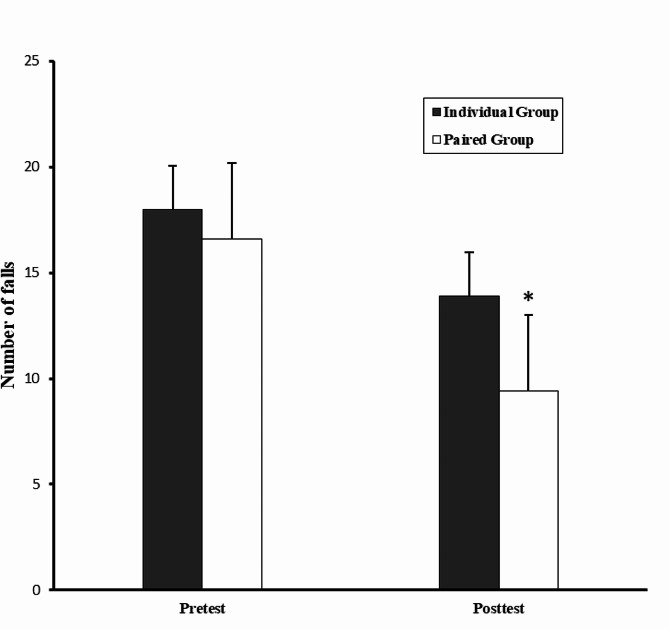
Comparison of the pre-test and post-test of the balance variable in the individual and paired groups. The asterisk indicates significant difference with the individual group

**Fig. 3:**
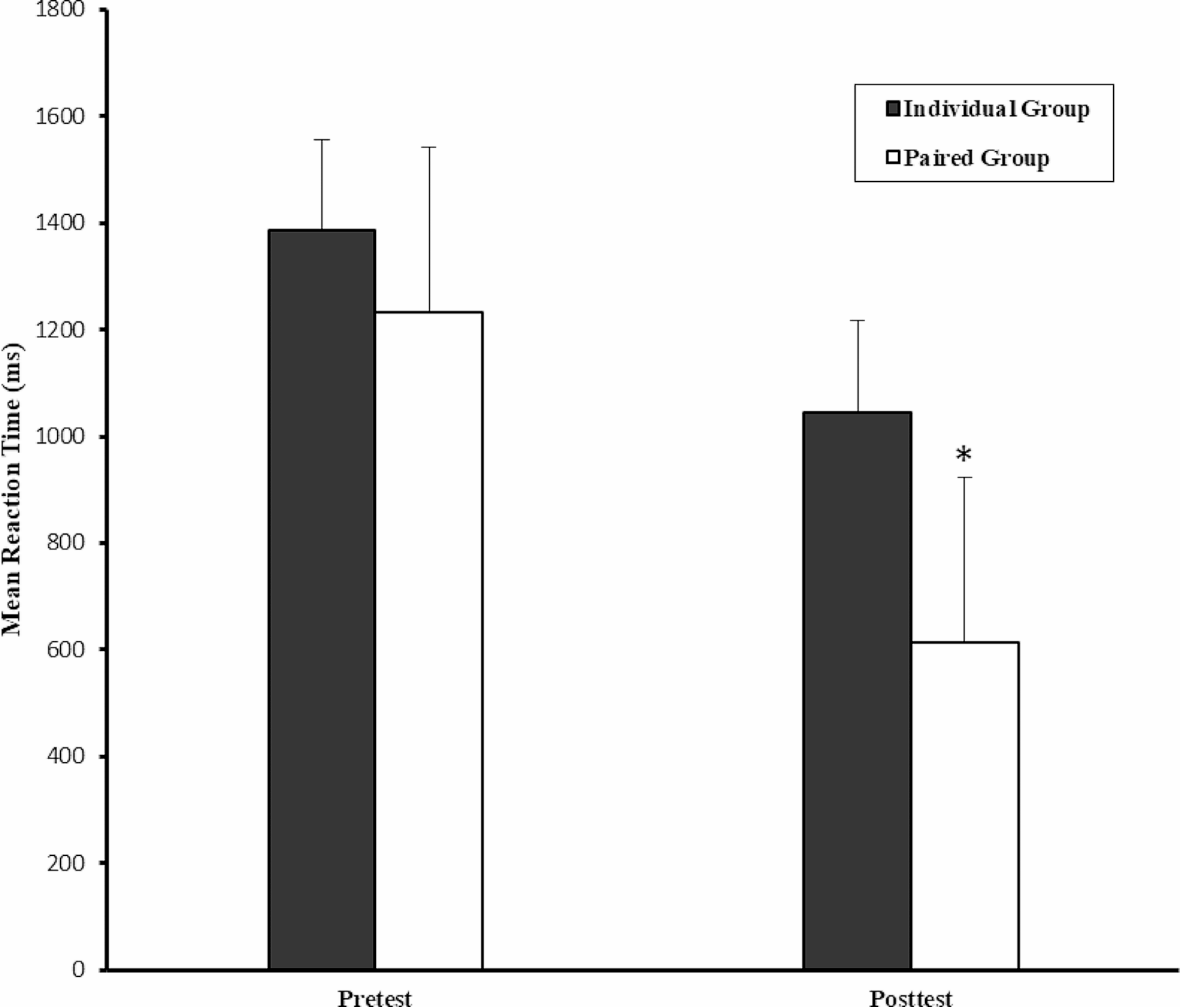
Comparison of the pre-test and post-test of the reaction time variable in the individual and paired groups. The asterisk indicates significant difference with the individual group

The comparison of paired and individual groups in the post-test showed that the performance of the paired group was significantly better in the balance variable (*p* = 0.01) and in the reaction time variable (*p* = 0.001). Figure 2 shows the performance of the groups in the balance variable and Fig. 3 shows the performance of the groups in the reaction time variable in the post-test.

In Fig. 2, balance scores indicate the number of times a person loses his balance during the test (60 s). As such, the type of balance test is descending, and in the post-test, the individual and paired groups have improved as the number of times they have lost their balance decreased.

In Fig. 3, reaction time scores indicate the average time (in milliseconds) that the participants have obtained in the reaction time test. As such, this test is also of the descending type, and in the post-test, the individual and paired groups have improved and their reaction time has decreased.

## Discussion

Various studies have shown that children with ID often have perceptual-motor disabilities as well [24], so it is possible to provide exercises aimed at reducing perceptual-motor disabilities for children with ID. The purpose of this research was to investigate the effect of individual and paired Brailletonik exercises on the balance and reaction time of children with ID. Participants were divided into individual and paired groups. The pre-test was conducted with the Stork Stand Test and simple reaction time software. Then 21 training sessions were held in 7 weeks and three sessions per week. After the training sessions, the post-test was taken and the scores of the participants were recorded. The results showed that children in the paired group had better performance in balance and reaction time than the individual group. In general, this study showed that a course of Brailletonik exercise is effective on the balance and reaction time variables.

The result obtained from this research about the effect of physical activity on balance is consistent with the results of many research. Yilmaz et al. [25] and Kubilay et al. [26] in their research on children with ID concluded that exercise programs were useful for the balance of these children. Jankowicz-Szymanska et al. [27] investigated the effect of physical training on static balance in young people with ID. The participants underwent a three-month sensorimotor training program. After the training sessions, the results of the tests were improved in the group of people under the training program. However, the difference between the groups was not statistically significant, and it can be concluded that the training improves deep sensibility in individuals with mild ID. Cuesta-Vargas et al. [28] also found physical training to be ineffective on the balance of adults with ID, which was inconsistent with the present study. The reason for this discrepancy was the type of exercise, the intensity of the exercise, the intervention protocol, and the duration of the exercise. Balance problems are very noticeable in individuals with disorders. Studies showed that these children had more problems maintaining balance than normally developing children, and this difference increased when visual information was removed, which was due to the importance of visual information that is created to control balance [29]. The ability to maintain balance is almost mandatory for the successful performance of all daily tasks and researchers have pointed out the role of different body systems in this case. The ability to maintain and control the posture of the body in space is the result of a complex interaction that occurs between different muscular, skeletal, and nervous systems, and the importance of each system varies according to its purpose in performing movement and different environmental conditions. In this model, the central nervous system is informed about the posture of the body’s center of gravity concerning gravity and the conditions of the base of support by using information from the visual, vestibular, and proprioceptive systems, and provides the appropriate response in the form of pre-programmed motor patterns [30]. Given that Brailletonik exercises are performed with the help of a six-house board, the participant can enter each house with a pair of legs or a single leg; also, in the intervention sessions, some exercises were performed with eyes closed, which aimed to strengthen balance in these children.

Regarding the effect of physical activity on reaction time, Un and Erbahçeci [31] reported that mentally-retarded children who participate in sports have a shorter reaction time than their sedentary counterparts. The results of the present study showed that Brailletonik exercises individually and in pairs were effective on the reaction time of children with ID. The effect of physical activities has also been mentioned in previous research. For example, Nareshkannan & Shelvam [32] conducted a study with the aim of investigating changes in flexibility and reaction time after twelve weeks of physical training in special children. To this end, 30 children between the ages of 13 and 19 were randomly selected and studied. The results of the study showed changes in the flexibility and reaction time of special children after twelve weeks of noticeable physical exercises for special children (42). Affes et al. [33] also conducted a study to examine the effect of low and moderate-intensity exercises on the reaction time and working memory of individuals with ID. The results showed that low and moderate- intensity exercises were effective on simple and choice reaction time as well as working memory, but moderate-intensity running was more suitable for strengthening auditory reaction time. Although physical activity and sports do not affect the intellectual state of mentally retarded children, it improved adaptive behavior and was important in the socialization of these children [3]. Therefore, motor learning and cognitive development had a vital relationship with each other, and primary motor learning was effective in the growth of brain cells, and hence its importance had to be emphasized [34].

Selective attention to some stimuli or failure to respond in time to others is often due to the insufficient capacity of the pathway or our inability to simultaneously address all the sensory guidance. following the repeated use of nerve pathways that resulted in practicing motor skills, the increase of nerve branches, and the formation of new synapses; the choice response capability increases [3]. Research has shown that reaction times in children with disorders are slower than their normal peers. The reaction time consists of two parts: premotor time and movement time. During the premotor time, cognitive or perceptual processing of the stimulus information is done, while during the movement time, the motor output of the response begins, and this slowness of the reaction time is due to premotor time or lack of motor planning. The type of intervention and the type of exercise performance mechanism are effective in reaction time. In disciplines such as Brailletonik, the reaction time may decrease due to the type of exercises provided.

In relation to the comparison between paired and individual groups, the results showed that paired Brailletonik exercises were more effective than individual Brailletonik exercises on balance and reaction time. As mentioned in the introduction, in paired exercises, learners experience positive mutual relationships and help each other to achieve the goal. The advantages of paired exercises have also been mentioned in previous research. For example, observational learning is one of the important benefits of paired practice, and conversation between learners is a useful opportunity for learning [35]. Also, practicing with another person in an interactive manner may increase learners’ motivation by adding a competitive component to the practice situation, and this condition may encourage learners to set more difficult goals [36]. In general, the results of this study showed that paired Brailletonik exercises had a greater effect on balance and reaction time than individual Brailletonik exercises. The limitations of this research included the small sample size, the lack of control over the amount of sleep, noise in school and the lack of assessment of the retention phase during research process.

## Conclusion

In general, the results showed that a course of Brailletonik exercise was effective on the balance and reaction time variables and children in the paired group had better performance than the individual group. Considering that Brailletonik exercises did not require special facilities and costs, and their implementation was not limited to a special space and environment, it was suggested that these exercise protocols be included as part of the exercise program for children with disabilities in special schools or sports centers. Finally, it was suggested to investigate the effect of Brailletonik exercise in other disorders such as autism, children with attention-deficit/hyperactivity disorder, and individuals with Down syndrome.

## Data Availability

The original contributions presented in the study are included in the article, further inquiries can be directed to the corresponding author.
